# Biolinguistic graph fusion model for circRNA–miRNA association prediction

**DOI:** 10.1093/bib/bbae058

**Published:** 2024-02-28

**Authors:** Lu-Xiang Guo, Lei Wang, Zhu-Hong You, Chang-Qing Yu, Meng-Lei Hu, Bo-Wei Zhao, Yang Li

**Affiliations:** School of Computer Science and Technology, China University of Mining and Technology, Xuzhou, 221116, China; School of Computer Science and Technology, China University of Mining and Technology, Xuzhou, 221116, China; Big Data and Intelligent Computing Research Center, Guangxi Academy of Sciences, Nanning 530007, China; College of Information Science and Engineering, Zaozhuang University, Shandong 277100, China; School of Computer Science, Northwestern Polytechnical University, Xi’an, 710129, China; College of Information Engineering, Xijing University, Xi’an 710123, China; School of Medicine, Peking University, Beijing, 100091, China; Xinjiang Technical Institute of Physics and Chemistry, Chinese Academy of Sciences, Urumqi 830011, China; School of Computer Science and Information Engineering, Hefei University of Technology, Hefei 230601, China

**Keywords:** CircRNA, MiRNA, circRNA–miRNA association, large-scale information network embedding, graph factorization, gradient boosting decision tree

## Abstract

Emerging clinical evidence suggests that sophisticated associations with circular ribonucleic acids (RNAs) (circRNAs) and microRNAs (miRNAs) are a critical regulatory factor of various pathological processes and play a critical role in most intricate human diseases. Nonetheless, the above correlations via wet experiments are error-prone and labor-intensive, and the underlying novel circRNA–miRNA association (CMA) has been validated by numerous existing computational methods that rely only on single correlation data. Considering the inadequacy of existing machine learning models, we propose a new model named BGF-CMAP, which combines the gradient boosting decision tree with natural language processing and graph embedding methods to infer associations between circRNAs and miRNAs. Specifically, BGF-CMAP extracts sequence attribute features and interaction behavior features by Word2vec and two homogeneous graph embedding algorithms, large-scale information network embedding and graph factorization, respectively. Multitudinous comprehensive experimental analysis revealed that BGF-CMAP successfully predicted the complex relationship between circRNAs and miRNAs with an accuracy of 82.90% and an area under receiver operating characteristic of 0.9075. Furthermore, 23 of the top 30 miRNA-associated circRNAs of the studies on data were confirmed in relevant experiences, showing that the BGF-CMAP model is superior to others. BGF-CMAP can serve as a helpful model to provide a scientific theoretical basis for the study of CMA prediction.

## INTRODUCTION

Although the being of circular ribonucleic acids (circRNAs) was first identified as early as the 1970s [1, 2], they have not been considered a crucial element in ribonucleic acid (RNA) expression analysis in human physiology. After 2013 [3, 4], a new batch of circRNAs was discovered and quantified due to high-resolution and high-throughput RNA-seq data, especially end-to-end reading and deep sequencing. According to the published literature, there are three types of identification methods for circRNAs: the method of ab initio prediction [3] based on RNA-seq comparison tools and algorithms, such as segemehl [5], and tools specifically designed to find circRNAs, such as CIRI [6]. These studies prove that circRNA is an endogenous, single-stranded, noncoding RNA (ncRNA) molecule with a covalent closed-loop structure generated as a back splicing linkage at the downstream 5′ end splice site and the upstream 3′ of RNA transcripts [3, 7], and it has vital biological functions, and treatment of diseases are critical by the identification of circRNA–disease associations [8–10]. Massive experimental analysis has shown that microRNA (miRNA) is an ncRNA and is an indispensable part of gene regulation in eukaryotes [11–13]. Up to now, increasing plentiful evidence reveals that circRNAs are miRNA sponges [14, 15], which can be used as a new class of biomarker through some biological experiments validation [16–18]. Despite this fact, exploring circRNA–miRNA interactions through some wet-lab experiments is commonly labor-intensive. To alleviate this trouble, plenty of simulation methods have been employed to speed up the identification of circRNA–miRNA associations (CMAs) [19, 20].

Recent research has revealed that rapidly spring-up computer algorithm models have provided well-grounded solutions for CMA prediction, realizing higher accuracy (Acc.) while working with huge data in machine learning technologies quickly. For instance, Lan *et al.* [21] put forward a NECMA model to predict CMAs using the inner product and neighborhood regularization logic matrix decomposition. Qian *et al*. [22] developed a CMIVGSD model based on singular value decomposition and graph variational auto-encoders to predict miRNA-associated circRNAs. Guo *et al*. [19] designed WSCD, which uses convolutional neural network and deep neural network (DNN) to predict CMAs based on structural DNN embedding. Wang *et al*. [23] integrate node similarity to form feature fusion by multi-modal information to infer the scores between circRNA-associated miRNAs pairs.

Additionally, most pre-existing CMAs prediction models neglect completely correlated biological information contained in their sequences and their influence on miRNAs function and complex associations with circRNAs. Accordingly, the following certain restrictions are obviously worth addressing: (i) rational the feature fusion model fusion of multi-modal heterogeneous information to extract interaction between circRNAs and association with different miRNAs; (ii) appropriate sequences and interaction information strategies are considered to learn good representations and (iii) experiment cross-reactions and noise influences need to be resolved.

**Figure 1:**
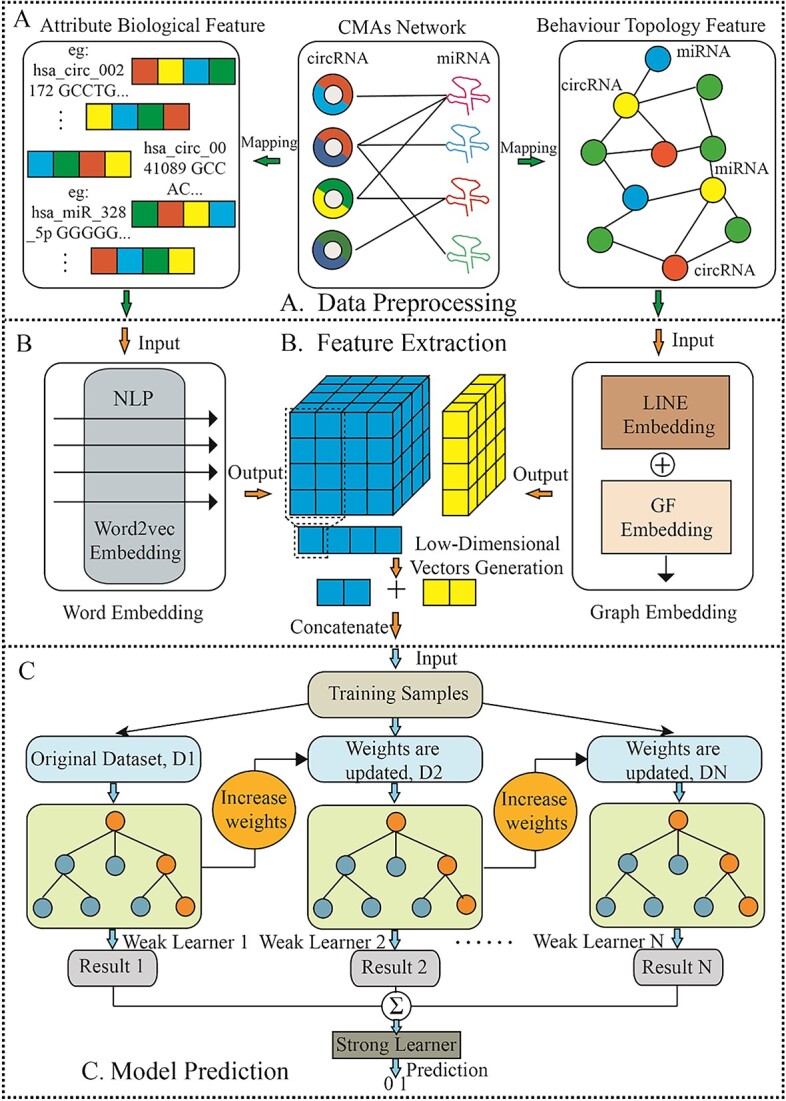
The workflow of BGF-CMAP model. **A.** Construction of nodes and nodes topology feature by pre-processing a heterogeneous association graph. **B.** The low dimensional representation vectors of circRNA and miRNA node sequences and topologies were learned by Word Embedding and Graph Embedding models. **C.** The flowchart of prediction by GBDT.

Encouraged by the above analysis consideration and the latest field correlation prediction research method [24–26], we put forward BGF-CMAP, a machine learning frame for predicting CMAs based on graph representation learning [27–29], which employs a homogeneous embedding fusion model of deep learning and factorization. To be specific, we first extract the potential biological attribute feature from the circRNA sequence-based word embedding, a natural language processing method, word2vec [30, 31]. Second, multi-source behavior feature information contains their impact on the circRNA–miRNA relationship of interaction [32], which are obtained as heterogeneous graphs by validated interaction pairs and the same number of unlabeled samples to construct a dependable molecular association network. Consequently, the network is input into a fusion model of large-scale information network embedding (LINE) [33, 34] and graph factorization (GF) [35] for low-dimensional embedding vector generation. Finally, gradient boosting decision tree (GBDT) [36] classifier is utilized to infer the potential CMAs effectively. In conclusion, the framework of the BGF-CMAP model is shown in Figure 1. Supplementary data are available online at https://github.com/look0012/BGF-CMAP.

## RESULTS

### Prediction performance of the BGF-CMAP model

**Figure 2:**
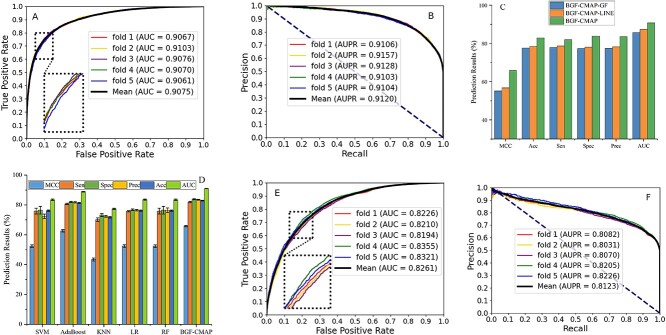
Performance evaluation of various models. (1) **A** and **B** show AUC and AUPR were achieved by our proposed BGF-CMAP. A. AUC was obtained by summing the areas under panel A plotting the ROC curve. **B.** AUPR refers to the area under the curve enclosed by precision and recall. (2) **C.** Comparison of prediction capability of diverse tactics. (3) **D.** Results of 5-fold CV acquired by different classifier models. (4) **E** and **F** show AUC and AUPR were achieved by the LAP method. E. ROC curves yielded by the results of LAP on the dataset using 5-fold CV. F. AUPR yielded by the results of LAP on the dataset using 5-fold CV.

In the evaluation, we used our constructed dataset using the 5-fold cross-validation (CV) method to access the ability of BGF-CMAP model to predict potential CMAs in terms of Acc., sensitivity (Sen.), specificity (Spe.), precision (Pre.), Matthews correlation coefficient (MCC), area under receiver operating characteristic (ROC) (AUC) and area under precision recall (AUPR). All experimental results and the mean of prediction in boldface are shown in Table 1. BGF-CMAP acquired a mean AUC of 0.9075 and an SD of 0.0016, of which the $\text{AUC}$s of 5-fold experiments were 0.9067, 0.9103, 0.9076, 0.9070 and 0.9061, respectively. In Figure 2, the $\text{AUC}$ of BGF-CMAP can be obtained by summing the areas under panel A plotting the $\text{ROC}$ curve, and the $\text{AUPR}$ of BGF-CMAP refers to the AU$\text{PR}$ curve enclosed by $\text{Pre}.$ and recall in panel B, reaching the upper left and right corner of the image with a large area under the curve, respectively. Overall, the above statistics prove that assuming the model has state-of-the-art performance, it can excellently provide solid evidence for an advanced understanding of the circRNA–miRNA relationship by effectively predicting potential CMAs.

**Table 1:** Results of 5-fold CV obtained by BGF-CMAP, BGF-CMAP-LINE and BGF-CMAP-GF

Model	5-fold	Acc. (%)	Sen. (%)	Spe. (%)	Pre. (%)	MCC (%)	AUC
BGF-CMAP	Fold 1	82.72	81.35	84.09	83.64	65.46	0.9067
	Fold 2	82.82	81.91	83.72	83.42	65.65	0.9103
	Fold 3	83.33	82.38	84.27	83.96	66.66	0.9076
	Fold 4	82.97	82.58	83.37	83.24	65.95	0.9070
	Fold 5	82.67	81.59	83.74	83.38	65.35	0.9061
	Average	82.90	81.96	83.84	83.53	65.81	0.9075
	SD	0.270	0.520	0.350	0.280	0.520	0.0016
BGF-CMAP–LINE	Fold 1	79.11	79.22	79.00	79.04	58.21	0.8736
	Fold 2	78.81	78.85	78.77	78.79	57.62	0.8777
	Fold 3	79.29	78.87	79.71	79.54	58.59	0.8794
	Fold 4	77.57	78.81	76.34	76.91	55.17	0.8683
	Fold 5	76.98	77.43	76.53	76.74	53.96	0.8676
	Average	78.35	78.64	78.07	78.20	56.71	0.8733
	SD	1.020	0.690	1.530	1.290	2.030	0.0053
BGF-CMAP–GF	Fold 1	76.58	76.60	76.57	76.58	53.17	0.8516
	Fold 2	77.96	78.35	77.56	77.74	55.91	0.8583
	Fold 3	77.62	78.28	76.97	77.26	55.25	0.8516
	Fold 4	77.77	77.75	77.78	77.77	55.53	0.8598
	Fold 5	77.93	78.42	77.43	77.65	55.86	0.8642
	Average	77.57	77.88	77.26	77.40	55.14	0.8571
	SD	0.570	0.760	0.490	0.500	1.140	0.0055

### Comparison of different feature extraction strategies

The purpose and advantage of combining sequence information with interaction information is to capture the linear and nonlinear relationship between features, which makes the model more robust and accurate. To confirm whether the effectiveness of two different graph embedding methods (LINE and GF) better than the proposed model to model performance, we contrasted testing LINE and GF models with BGF-CMAP, respectively, named BGF-CMAP-LINE and BGF-CMAP-GF. BGF-CMAP-LINE and BGF-CMAP-GF with BGF-CMAP performed the same 5-fold CV experiment on the same dataset, the comparison is summarized in the histogram in Figure 2C, and their specific values are shown in Table 1. According to the data in Table 1, the average values of $\text{Acc}.$, $\text{Sen}.$, $\text{Spe}.$, $\text{Pre}.$, $\text{MCC}$ and $\text{AUC}$ obtained from the BGF-CMAP–LINE model were 3.65, 3.32, 5.77, 5.33 and 9.1% and 0.0342 less than the presented model in this paper’s BGF-CMAP model, respectively. Comparing the models in Table 1 indicates that the average values of the BGF-CMAP–GF model are smaller than the built BGF-CMAP model. Similarly, Figure 2C also shows the advantage of BGF-CMAP through the comparison of prediction performance. In conclusion, the feature extraction effect of BGF-CMAP is better than that of the two unilateral information feature extraction strategies.

### Comparison with Laplacian eigenmaps model

To infer the effectiveness of the proposed model utilizing biological attribute feature and multi-source behavior feature as model attributes to optimize this model capability, we compared it with the behavior topology feature vector generated by the LAP method. For fairness and consistency, we used the method of low-dimensional embedding vectors generated by LAP to replace the graph embedding of the proposed fusion model during the experience process, and the other parts of the model remained unchanged. Specifically, we extract the interaction behavior features and sequence the attribute features using LAP and Word2vec, respectively, and then integrate these two features into the GBDT classifier for model training. Using our dataset, we train the model with the LAP method using the 5-fold CV to obtain the values summarized in Table 2. As seen from Table 2, BGF-CMAP achieved the better result, and its prediction $\text{Acc}.$, $\text{Spe}.$, $\text{Pre}.$, $\text{MCC}.$ and $\text{AUC}$ are higher than the LAP model by 8.91, 22.6, 14.4 and 16.18% and 0.0814, respectively. This result suggests that a fusion model combining LINE and GF used by the proposed model can effectively build the characterization of vectors and train the computational model, which contributes to improving the model and achieving the most potential prediction performance. The $\text{AUPR}$ and $\text{ROC}$ curve advantages of BGF-CMAP can be seen from the comparison of Figure 2E and F.

**Table 2:** Results of 5-fold CV obtained by LAP and BGF-CMAP

5-fold	Acc. (%)	Sen. (%)	Spe. (%)	Pre. (%)	MCC (%)
Fold 1	73.44	87.38	59.50	68.33	48.82
Fold 2	74.10	86.76	61.43	69.23	49.82
Fold 3	73.03	86.37	59.70	68.18	47.80
Fold 4	75.04	87.40	62.68	70.08	51.69
Fold 5	74.34	85.77	62.91	69.81	50.00
Average	73.99 ± 0.78	86.74 ± 0.69	61.24 ± 1.6	69.13 ± 0.85	49.63 ± 1.45
BGF-CMAP	82.90 ± 0.27	81.96 ± 0.52	83.84 ± 0.35	83.53 ± 0.28	65.81 ± 0.52

### Comparison with diverse classifier models

To ensure the selection of an optimal classification method through feature extraction, we compare diverse classifier models to assess the impact on the features and performance of BGF-CMAP in this study. Specifically, we retained the biological attribute feature and behavior topology feature extraction methods as unvaried and only substituted the GBDT model with five different classifiers, including K-nearest neighbor (KNN), logistic regression (LR), rotation forest (RF), support vector machine (SVM) and AdaBoost algorithm for research. Table 3 lists the mean values of the 5-fold CV experiments achieved by the above models on the same dataset, and it is also shown in bar Figure 2D. Summarizing from Table 3, AdaBoost achieved the second rank in $\text{Acc}.$, $\text{Sen}.$, $\text{Spe}.$, $\text{Pre}.$, $\text{MCC}$ and $\text{AUC}$, but it was 1.54, 1.26, 1.82, 1.76 and 3.09% and 0.0189 lower than the best BGF-CMAP model results. And the comparison in Figure 2D also shows that the BGF-CMAP model is the best result. In a nutshell, these results have demonstrated that our BGF-CMAP model by GFBDT classifier outperforms other classifier models.

**Table 3:** The mean results were performed by different classifiers model

Indicator	AdaBoost	KNN	LR	RF	SVM	BGF-CMAP
Acc. (%)	81.36 ± 0.43	71.69 ± 0.50	76.24 ± 0.45	76.17 ± 0.46	69.40 ± 0.81	82.90 ± 0.27
Sen. (%)	80.70 ± 0.46	70.14 ± 1.18	75.73 ± 0.46	75.88 ± 1.91	66.78 ± 1.00	81.96 ± 0.52
Spe. (%)	82.02 ± 0.46	73.24 ± 1.00	76.75 ± 0.54	76.47 ± 2.57	72.01 ± 1.04	83.84 ± 0.35
Pre. (%)	81.77 ± 0.45	72.39 ± 0.61	76.51 ± 0.49	76.38 ± 1.54	70.47 ± 0.90	83.53 ± 0.28
MCC (%)	62.72 ± 0.85	43.41 ± 1.0	52.48 ± 0.90	52.39 ± 0.92	38.85 ± 1.62	65.81 ± 0.52
AUC	0.8886 ± 0.0024	0.7736 ± 0.0045	0.8359 ± 0.0037	0.8349 ± 0.0048	0.7713 ± 0.0061	0.9075 ± 0.0016

**Table 4:** The AUC and AUPR scores generation of various models

Methods	CMIVGSD	WSCD	KGDCMI	SGCNCMI	BGF-CMAP
AUC	0.8804	0.8898	0.8930	0.8942	0.9075
AUPR	0.8629	0.8847	0.8767	0.8887	0.9120

### Comparison with other state-of-the-art models

As recent research on the CMAs has intensified, many distinguished scholars have proposed different approaches to predicting CMAs. We look forward to comparing BGF-CMAP with the above methods more equitably to evaluate its predictive performance. The proposed pipeline has many competitive advantages, as shown in Figure 1. Specifically, the biggest advantage of word2vec is to encode words with similar meanings into high-dimensional vectors with similar distances so that the encoding has semantic characteristics. GF uses approximate factorization of adjacency matrices as embedding. LINE extends this approach and attempts to maintain 1st and 2nd order approximations, aiming to map each node in the graph into a low-dimensional vector space, thereby capturing the similarities and relationships between nodes. The edge sampling method of LINE overcomes the problem of node embedding aggregation, which can easily occur in the traditional stochastic gradient method, and improves the efficiency and effect of the result [33]. Compared with our model, other models in this research field do not have the advantages of this model’s integration of the above algorithms, so they cannot show good competitive results. Because there is a precise comparison here, we counted the $\text{AUC}$ and $\text{AUPR}$ scores generated by the prior models, and these listed results in Table 4, containing our model and only several newly published papers in the new research field of CMAs prediction with CMIVGSD, WSCD, KGDCMI [23] and SGCNCMI [20]. The Table 4 shows BGF-CMAP realized the highest $\text{AUC}$ and $\text{AUPR}$ scores, which were 0.0133 and 0.0233 times superior to the second-best SGCNCMI model and exceeded the mean value of the other three methods by about 0.0198 and 0.0372 times. It can be deduced from Table 4 that the *P*-value of 0.0270 is <0.05, so it can be concluded that our BGF-CMAP has significant differences compared to other models. Therefore, from the above comparison, it can be concluded that BGF-CMAP can provide the most competitive theoretical guidance for further academic research.

### Case studies

**Figure 3:**
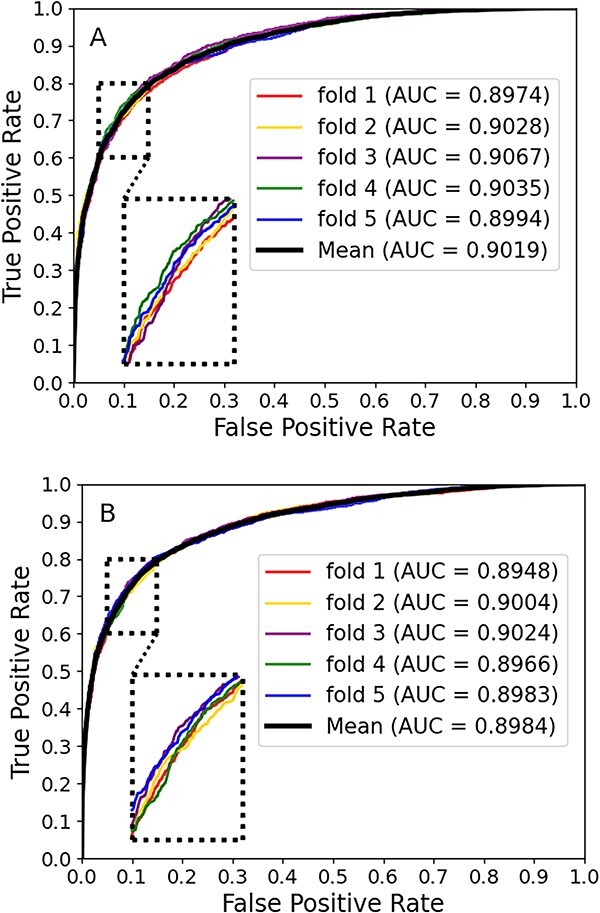
Panels (**A**) and (**B**) show that AUC was achieved by datasets CMI-9589 and CMI-9905, respectively.

To further research the effectiveness of BGF-CMAP to identify new miRNA candidate circRNAs, we conducted case studies by training the model with known circRNA–miRNA pairs and deduced all unknown CMAs with the trained model. Candidates for unknown interaction pairs are then ranked according to the higher scores, and the predictive validity is made sure it is correct by finding relevant research literature or correlation experiment. Concluding model prediction outcomes are presented in Table 5 from which we can see that only 7 of the top 30 miRNA-related circRNAs pairs have been not verified in the recent literature. In order to validate the reliability, robustness and accurate predictive ability of our model, we tested our BGF-CMAP model using the latest literature datasets CMI-9905 [23] and 9589 pairs of CMAs (called CMI-9589) [22]. The final experimental outcomes are shown in Figure 3. From Figure 3, it can be observed that our model achieved AUC values of 0.8984 and 0.9019, which fully show the strong applicability of our model. In addition, we conducted another case study on the model using an independent dataset mentioned above. The model was used to predict all unknown CMAs, and out of the top 10 scoring CMAs, 8 of the associated relationships have been successfully reported in relevant literature, as shown in Table 6. Overall, the case study suggests that BGF-CMAP has a superior predictive performance for the prediction of potential CMAs and that these valuable circRNA candidates for miRNA studies will most likely be selected for additional wet-lab experimental studies to reduce the deficiency of manual errors.

**Table 5:** 30 CMAs pairs predicted by BGF-CMAP

Rank	circRNA	miRNA	Evidence	Rank	circRNA	miRNA	Evidence
1	hsa_circ_0051922	hsa-miR-4739	Confirmed	16	hsa_circ_0013871	hsa-miR-612	Unconfirmed
2	hsa_circ_0051285	hsa-miR-4739	Confirmed	17	hsa_circ_0013871	hsa-miR-1273h-5p	Unconfirmed
3	hsa_circ_0039128	hsa-miR-4739	Confirmed	18	hsa_circ_0061080	hsa-miR-6860	Confirmed
4	hsa_circ_0081673	hsa-miR-612	Confirmed	19	hsa_circ_0010663	hsa-miR-3187-5p	Confirmed
5	hsa_circ_0055327	hsa-miR-612	Confirmed	20	hsa_circ_0048709	hsa-miR-612	Confirmed
6	hsa_circ_0080666	hsa-miR-4739	Confirmed	21	hsa_circ_0039087	hsa-miR-612	Unconfirmed
7	hsa_circ_0013876	hsa-miR-612	Unconfirmed	22	hsa_circ_0009652	hsa-miR-612	Confirmed
8	hsa_circ_0081678	hsa-miR-346	Confirmed	23	hsa_circ_0081678	hsa-miR-3187-5p	Confirmed
9	hsa_circ_0081678	hsa-miR-4739	Confirmed	24	hsa_circ_0010541	hsa-miR-346	Unconfirmed
10	hsa_circ_0082878	hsa-miR-4739	Confirmed	25	hsa_circ_0085900	hsa-miR-3187-5p	Confirmed
11	hsa_circ_0081673	hsa-miR-6860	Confirmed	26	hsa_circ_0010676	hsa-miR-6860	Confirmed
12	hsa_circ_0065481	hsa-miR-612	Unconfirmed	27	hsa_circ_0005266	hsa-miR-612	Confirmed
13	hsa_circ_0055327	hsa-miR-6860	Confirmed	28	hsa_circ_0055327	hsa-miR-4739	Confirmed
14	hsa_circ_0048709	hsa-miR-6860	Confirmed	29	hsa_circ_0039087	hsa-miR-4739	Confirmed
15	hsa_circ_0020490	hsa-miR-3187-5p	Confirmed	30	hsa_circ_0065481	hsa-miR-6860	Unconfirmed

**Table 6:** 10 CMAs pairs predicted by BGF-CMAP

Rank	circRNA	miRNA	PMID	Rank	circRNA	miRNA	PMID
1	hsa_circ_0001946	hsa-miR-7-5p	33326806	6	hsa_circ_0000073	hsa-miR-1184	37506860
2	hsa_circ_0000218	hsa-miR-139-3p	35185288	7	hsa_circ_0107593	hsa-miR-20a-5p	33585208
3	hsa_circ_0139402	hsa-miR-326	35154515	8	hsa_circ_0107593	hsa-miR-93-5p	33585208
4	hsa_circ_0004812	hsa-miR-1287-5p	Unconfirmed	9	hsa_circ_0107593	hsa-miR-106b-5p	33585208
5	hsa_circ_0001947	hsa-miR-329-5p	32657138	10	hsa_circ_0001079	hsa-miR-136-5p	Unconfirmed

## DISCUSSION

In this paper, we proposed BGF-CMAP, a graph representation learning model based on a fusion model combining two homogeneous embedding methods for predicting CMAs. We first constructed low-dimensional embedding vector generation based on word embedding using the sequence information and then constructed a low-dimensional representation based on the fusion model combining LINE and GF, while retaining graph topology and attributes of nodes. Next, we got the training features of the fusion vectors by combining the biological attribute features and multi-source behavior feature information to learn the interaction scores of circRNAs with miRNA in the feature latent interactions. Overall assessments of the built datasets confirmed that BGF-CMAP is superior to other marvelous models in CMAs prediction. To evaluate the optimal capability of this paper, we carried out several ablation experiments on the same datasets, containing a comparison of the BGF-CMAP–LINE model, BGF-CMAP–GF and LAP models, different experimental classifier models and comparison of association prediction model performance with additional leading-edge models. Overall assessments of the built datasets confirmed that BGF-CMAP is superior to other cutting-edge models in CMA prediction. Prediction of numerous experimental results pairs obtained higher association scores in case studies in the recent literature. A careful analysis of the results indicates that our proposed model achieved an excellent prediction of potential CMAs for academic research.

The reasons for the excellent predictive performance of the BGF-CMAP model in this paper are due to several advantages, which are described in the following text. Especially, the model takes into consideration the attribute features and behavior features provided by circRNA and miRNA sequences, whereas previous approaches have focused less on the full application of this information. More specifically, (i) comparing with the previous method, which focuses on the CMA feature extraction information, the sequence information is used to enhance the feature expression. (ii) It can execute genome-wide quantitative testing and analysis, and virtually every reading segment can well characterize its correlation RNA sequence, and there are no experiment cross-reactions and noise influences. Furthermore, the sequence information is combined with the interaction information to obtain extremely reliable feature vectors of behavior attributes and to increase the quality of feature extraction.

Nevertheless, the following certain restrictions of the BGF-CMAP model are certainly worth resolving in the future. For instance, (i) sequence information is not wholly employed by the proposed model, and the characteristics part of its additional information hiding needs to be studied ulteriorly. (ii) The application of this framework requires parameter settings, which possibly leads to some minor simulation errors compared with the actual experiment. (iii) Explore more on the mechanism of perfect multi-source heterogeneous fusion. Combined with the above discussion, we intend to research large feature dimensions and multi-source heterogeneous fusion model to make unremitting efforts to conduct perfectly the CMAs prediction model.

## MATERIALS AND METHODS

### Dataset

To use high-reliability sequence information of circRNA and miRNA to assess the performance of the BGF-CMAP model, we utilized the currently available experimentally verified CircBank database [37] and miRbase [38, 39] database as the high-quality selected dataset to assess the quality of the above model. CircBank utilizes circRNA data from the circBase database (http://www.circbase.org/), encompassing information for comprehensive analysis and processing, especially the complete circular RNA sequence on its website. It offers circRNA annotation details, mature sequences, protein-coding potential, IRES prediction, m^6^A modification status, circRNA conservation and miRNA-circRNA interaction information, among other features. First, we removed the redundancy of high-quality CMAs from the CircBank database, and second, we extracted CMAs from published journals, checked their names and sequences against standard circBase and miRbase databases and finally merged them into our constructed database. Therefore, we can describe the built dataset as follows:


(1) \begin{equation*} D={D}^{+}\cup{D}^{-}. \end{equation*}

In this study, we first clarified 20 208 pairs of experimentally validated CMAs involving 3569 circRNAs and 1152 miRNAs from the built dataset. Here, ${D}^{+}$ and ${D}^{-}$ denote the set of 20 208 positive and 4 091 280 negative samples, respectively. $D$ denotes a union set between elements. Second, we established the adjacent matrix $DM$ of the built dataset with the dimension of 3569 × 1152. When miRNA $m(i)$ is not associated with circRNA $c(j)$, the element $DM\left(i,j\right)$ of $DM$ is set to 0; Otherwise, it is given a value of 1.

### Evaluation criteria

For the evaluation performed in machine learning, different evaluation metrics to assess the prediction capability of the proposed BGF-CMAP model, including $\text{Spe}.$, $\text{Pre}.$, $\text{Sen}.$, $\text{MCC}$ and $\text{Acc}.$ These evaluation indices are defined as follows:


(2) \begin{equation*} \text{Spe}.=\frac{\text{TN}}{\text{TN}+\text{FP}}, \end{equation*}

(3) \begin{equation*} \text{Pre}.=\frac{\text{TP}}{\text{TP}+\text{FP}}, \end{equation*}

(4) \begin{equation*} \text{Sen}.=\frac{\text{TP}}{\text{TP}+\text{FN}}, \end{equation*}

(5) \begin{equation*} \text{MCC}=\frac{\text{TP}\times \text{TN}-\text{FP}\times \text{FN}}{\sqrt{\left(\text{TP}+\text{FP}\right)\left(\text{TP}+\text{FN}\right)\left(\text{TN}+\text{FP}\right)\left(\text{TN}+\text{FN}\right)}}, \end{equation*}

(6) \begin{equation*} \text{Acc}.=\frac{\text{TP}+\text{TN}}{\text{TP}+\text{TN}+\text{FP}+\text{FN}}, \end{equation*}

where the abbreviations of the evaluation criteria include $\text{TP}$ for true positive, $\text{TN}$ for true negative, $\text{FP}$ for false positive and $\text{FN}$ for false negative. Additionally, we visualized the $\text{ROC}$ curves by computing the $\text{TP}$ and $\text{FP}$ rates that were generated from BGF-CMAP and calculated their average $\text{AUC}$, $\text{AUPR}$ for considering the imbalance. Also, a reliable 5-fold CV was applied to reduce the overfitting and to assess the performance of our proposed BGF-CMAP.

### Attribute feature extraction by Word2vec

The word2vec was proposed by Google [30]. The prevailed word2vec is a word embedding model, which uses the relationship between the words appearing in the sentences to get word vectors from higher dimensional to lower dimensional space in machine learning [40, 41]. Analogously, a node is used as a word and a sequence of nodes is used as a sentence, as described in the sequence analysis of the circRNA and miRNA node vector representation by the word2vec algorithm.

Here, word2vec made use of the extracted circRNA and miRNA sequence features in a vector space dependent on CBOW (bag-of-words) [24, 42] model that is utilized to characterize the node features in this study. The optimal target word determined by CBOW performs $n$ predictions before and after the target word, with the objective function of the output value of CBOW, as follows:


(7) \begin{equation*} {J}_{\theta }=\frac{1}{T}\sum_{t=1}^T\log p\left({\omega}_t\left|{\omega}_{t-n},\cdots, {\omega}_{t-1},{\omega}_{t+1},\cdots, {\omega}_{t+n}\right.\right), \end{equation*}

where $\omega$ is weight matrix, ${\omega}_t$ stands for the target word and parameters of the context words are regarded as ${\omega}_{t-n},\cdots, {\omega}_{t+n}$.

In the study, through the gensim python software package [43], using the word2vec model, which trains the circRNA and miRNA sequence vectors, we achieved a 64-dimensional objective vector.

### Behavior feature extraction by homogeneous graph embedding

Homogeneous graph embedding [44, 45] is a graph representation learning method, which aims to retain graph topology when learning low-dimensional representations of vertices. It is also acknowledged as network embedding or non-attributed graph embedding containing methods based on random walks, deep learning and matrix decomposition. Therefore, GF, LAP in matrix factorization and LINE of deep learning are selected for this study.

The widely accepted network representation learning model (LINE [46]) can be considered as learning low-dimensional dense vectors by preserving 1st and 2nd order proximity using a multi-layer perceptron. For objective functions of 1st and 2nd orders, respectively, are shown below:


(8) \begin{equation*} {O}_1=d\left({\hat{p}}_1\left(\cdot, \cdot \right),{p}_1\left(\cdot, \cdot \right)\right), \end{equation*}

(9) \begin{equation*} {O}_2=\sum_{v_i\in V}{\lambda}_id\left({\hat{p}}_2\left(\cdot |{v}_i\right),{p}_1\left(\cdot |{v}_i\right)\right). \end{equation*}

Here, we set *V* = {${v}_i,L,{v}_n\Big\}$ and $E={\left\{{e}_{ij}\right\}}_{ij=1}^n$ to denote vertices and edges, respectively, which means ${e}_{i,j}$ goes from ${v}_i$ to ${v}_j$, in graph $G\left(V,E\right)$; $d\left(\cdot, \cdot \right)$ represents the distance between two distributions; ${\hat{P}}_1\left(\cdot, \cdot \right)$ and ${\hat{P}}_2\left(\cdot |{v}_i\right)$ are regarded as its representation and the empirical distribution, and ${P}_1\left(\cdot, \cdot \right)$ and ${P}_1\left(\cdot |{v}_i\right)$ stand for joint and context conditional distribution, respectively.

To solve the storage and the construction for the downstream classifier, GF is selected to factorize the adjacent matrix of the graph. This object function is described as


(10) \begin{equation*} f\left(Y,Z,\lambda \right)=\frac{1}{2}\sum_{\left(i,j\right)\in E}{\left({Y}_{ij}-\left\langle{Z}_i,{Z}_j\right\rangle \right)}^2+\frac{\lambda }{2}\sum_i{\left\Vert{Z}_i\right\Vert}^2 \end{equation*}

where $Y,Z$ and $\lambda$ denote the weight adjacency matrix, the factor matrix and the regularization parameter, respectively.

**Figure 4:**
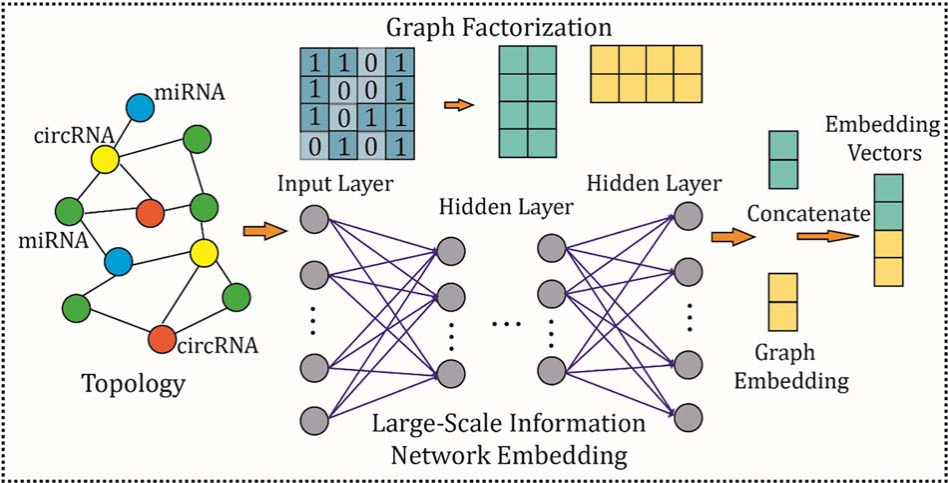
A homogeneous embedding fusion model.

In the operations of behavior feature extraction, combining the advantages of deep learning and factorization, a fusion graph embedding model based on ensemble LINE and GF is proposed, which is shown in Figure 4.

To compare whether the fusion model (BGF-CMAP) is convincing, we propose model LAP, a matrix factorization aiming to factorize high- the dimensional matrix to obtain the embeddings. In particular, the obtained objective function is minimized by Laplacian eigenmaps [47].


(11) \begin{equation*} \varphi (Y)=\frac{1}{2}\sum_{i,j}{\left|{Y}_i-{Y}_j\right|}^2{W}_{ij}= tr\left({Y}^T LY\right), \end{equation*}

where ${Y}_i$ and ${Y}_j$ are the embeddings of a node and the embedding of a graph denoted by $Y$; ${W}_{ij}$ and $L$ are regarded as its representation weight and the Laplacian of the graph $G$, respectively.

In this paper, the built open-NE library is applied to carry out the behavioral feature extraction of the graph embedding model. After training the model described above, we obtain the results with 64 dimensions per node.

### GBDT classifier for prediction

Jerome H. Friedman put forward GBDT [48–50], which is one of the best decision tree-based algorithms that be used either for categorization or regression or for filtering features. Under categorization strategy, through multiple iterations and the residual of the previous classifier, the GBDT model can be better to get a weak classifier. We can express the loss function of the weak classifier as


(12) \begin{equation*} {\hat{\theta}}_m=\underset{\theta_m}{\text{argmin}}\sum_{i=1}^NL\left({y}_i,{F}_{m-1}\left({x}_i\right)+T\left({x}_i;{\theta}_m\right)\right). \end{equation*}

Here, ${F}_{m-1}(x)$ represents the iteration result at this point, and we set the weak classifier (a decision tree) to the parameter value of $T\left({x}_i;{\theta}_m\right)$ and methods; the number of iterations is denoted by $m$.

We can see that the main computational costs of our proposed model are the feature extraction phase using graph embedding and the training stage of the GBDT classifier. First, in the graph embedding feature extraction stage, the time complexity of both LINE and GF can be approximately regarded as $O\left(\left|E\right|d\right)$, where $\left|E\right|$ shows the number of edges in a graph and d shows the embedding dimension of each node. Specifically, the time complexity $O\left(\left|E\right|d\right)$ indicates that the algorithm roughly requires $E\ast d$ computational operations during the calculation process. Second, in the GBDT training stage, the time complexity is as follows: the time complexity of constructing each decision tree is $O\left( Nf\log (N)\right)$, where $N$ is the number of samples and *f* is the number of features. GBDT usually iterates multiple rounds, and each round constructs a decision tree. Hence, the overall time complexity of the GBDT training stage is approximately $O\left( TNf\log (N)\right)$, where $T$ is the number of iterations. Therefore, the total time complexity of BGF-CMAP can be approximated as the sum of $O\left(\left|E\right|d\right)$ and $O\left( TNf\log (N)\right)$, which is approximately equal to $O\left( TNf\log (N)\right)$. Additionally, in terms of space complexity, the space complexity of graph embedding methods LINE and GF is relatively low and is mainly determined by the storage space of nodes, edges and adjacency lists. On the other hand, the space complexity of the GBDT model is usually high, especially when the dataset is large, the deepness of decision trees is deep and the number of leaf nodes is large, the memory space required by the model will increase accordingly. Therefore, we prioritize considering the space complexity of GBDT as the approximate space complexity of the model. After reasonable parameter tuning, in our work, the model algorithm was implemented and run using PyCharm Community Edition 2021.1 × 64, and the server was equipped with Intel(R) Core (TM) i7-12700H CPU and 16GB RAM. Ultimately, the average time for the training and prediction stages of running the GBDT classifier on a computer is approximately 16 min 21 s.

**Key Points**

- The complex association between circRNA and miRNA is a key regulatory factor in various pathological processes and plays a key role in most complex human diseases.
- Merging multiple information, incorporating graph representation learning (LINE and GF) and deep learning (CBOW) to more sufficiently extract attribute features and behavior features provided by circRNA and miRNA sequences than previous approaches.
- We proposed BGF-CMAP, which has adopted a homogeneous embedding fusion model and word2vec to train the model by fully extracting the interaction features combined with sequence information to predict CMAs.
- Abundant prediction experiments are adequate to demonstrate the dependability of the model, and the comparison can be concluded that our model can provide further academic research.

## Data Availability

The data can be freely downloaded from https://github.com/look0012/BGF-CMAP.

## Author Biographies

**Lu-Xiang Guo** is a PhD candidate at the China University of Mining and Technology. His research interests include bioinformatics, machine learning and complex network.

**Lei Wang** is a professor at the China University of Mining and Technology. His research interests include data mining, machine learning, deep learning, computational biology and bioinformatics.

**Zhu-Hong You** is a professor at the Northwestern Polytechnical University. His research interests include neural networks, intelligent information processing, sparse representation and its applications in bioinformatics.

**Chang-Qing Yu** is a professor at the Xijing University. His research interests include neural networks, data analysis and Internet of Things.

**Meng-lei Hu** is a master’s degree candidate at the Peking University. Her research interests include the integration of traditional Chinese and Western medicine, bioinformatics and machine learning.

**Bo-Wei Zhao** is a PhD candidate at the University of Chinese Academy of Sciences. His research interests include machine learning, complex networks and graph neural network.

**Yang Li** is a PhD candidate at the Hefei University of Technology, Hefei, China. His current research interests include machine learning, data mining and its applications in bioinformatics.
